# Neoplastic Disorders and Cardiovascular Comorbidities in Geriatric Patients: A Simple Association?

**DOI:** 10.3390/geriatrics11020035

**Published:** 2026-03-27

**Authors:** Andreea Taisia Tiron, Marian-Vlad Lăpădat, Maria Mădălina Georgică, Lavinia Alice Bălăceanu, Ion Daniel Baboi, Ion Dina

**Affiliations:** 1 Clinical Department 1-Medical Semiology, “Carol Davila” University of Medicine and Pharmacy, 020021 Bucharest, Romania; andreea.tiron@umfcd.ro (A.T.T.); marian-vlad.lapadat@drd.umfcd.ro (M.-V.L.); alice.balaceanu@umfcd.ro (L.A.B.); ion.dina@umfcd.ro (I.D.); 2 Cardiology Department, Clinical and Emergency Hospital “Sf. Ioan”, 042122 Bucharest, Romania; maria-madalina.georgica0825@rez.umfcd.ro; 3 Gastroenterology Department, Clinical and Emergency Hospital “Sf. Ioan”, 042122 Bucharest, Romania; 4 Internal Medicine Department, Clinical and Emergency Hospital “Sf. Ioan”, 042122 Bucharest, Romania

**Keywords:** cardiovascular disease, cancer, neoplastic disease, older patients, comorbidities in the geriatric population

## Abstract

Cardiovascular disease (CVD) and cancer frequently coexist in older patients, posing significant challenges in clinical management due to overlapping risk factors and treatment-related complications. This narrative review summarizes current knowledge on the epidemiology, shared pathophysiological mechanisms and clinical impact of neoplastic comorbidities in older adults with cardiovascular diseases. It highlights the increased mortality, morbidity and diminished quality of life resulting from the coexistence of these conditions. The review also discusses personalized management strategies, emphasizing comprehensive geriatric and cardiac assessments, and tailoring oncologic treatments to minimize cardiotoxicity, as well as the role of prevention and rehabilitation programs. As the population ages and cancer survival improves, integrated cardio-oncology care adapted to older adults becomes increasingly essential to optimize outcomes and preserve functional status.

## 1. Introduction

The coexistence of neoplastic diseases and cardiovascular diseases (CVDs) in older adults represents a complex and increasingly common clinical challenge. As populations age and medical care improves, more individuals live long enough to develop both conditions. CVD encompasses a wide range of disorders, including coronary artery disease, cerebrovascular disease, heart failure, arrhythmias, hypertension, peripheral arterial disease, rheumatic heart disease, congenital heart disease, deep vein thrombosis, and pulmonary embolism.

This overlap raises multiple issues. Shared risk factors—such as age, smoking, diabetes, and chronic inflammation—may predispose individuals to both cancer and CVD. Cancer therapies, including chemotherapy, radiotherapy, targeted therapy, and immunotherapy, can have cardiotoxic effects; these are particularly relevant in older patients whose cardiovascular reserve is already reduced. The presence of CVD may limit or complicate cancer treatment options through dose reductions or the avoidance of certain agents. Conversely, the burden of cancer—including systemic effects, cachexia, and metabolic changes—may aggravate cardiovascular disease. Older patients often have reduced physiological reserves, multiple comorbidities, polypharmacy and increased vulnerability (frailty), all of which influence prognosis, treatment tolerability, and outcomes.

Many geriatric patients are diagnosed with cancer late due to delayed symptom recognition or a refusal of/delay to seeking treatment, often stemming from age-related beliefs or cognitive impairment. Consequently, diagnosis frequently occurs at an advanced stage, leading to increased complications from surgical and pharmacological interventions [1,2].

Older adult patients are particularly vulnerable due to frailty, polypharmacy, multimorbidity, and reduced organ reserve, all of which complicate therapeutic decisions and affect treatment tolerance. This creates significant clinical complexity: cancer therapies can exacerbate cardiovascular conditions through direct or indirect cardiotoxic effects, while pre-existing cardiovascular diseases can limit cancer treatment options, delay diagnosis, or worsen prognosis. In this context, understanding the interplay between oncology and cardiology in geriatric patients is essential for improving clinical outcomes and quality of life [3].

The aim of this narrative review is to provide an updated overview of the prevalence, shared pathophysiological mechanisms, clinical implications, and management challenges of neoplastic comorbidities in geriatric patients with cardiovascular disease, while also highlighting existing gaps in knowledge and opportunities for future research.

Therefore, studying neoplastic comorbidities in older patients with CVD is important from both scientific and practical standpoints. This research helps guide decision-making, optimize therapy, monitor and prevent complications, and improve survival and quality of life.

## 2. Materials and Methods

A comprehensive literature search was conducted using Web of Science (Clarivate Analytics, Philadelphia, PA, USA), PubMed, and the websites of international clinical guidelines. A preliminary search was performed to identify relevant search terms in English and other languages known to the authors. We limited our selection to research published within ten years, between January 2016 and January 2026, to maintain a focus on contemporary clinical data; however, earlier studies were considered when relevant. The search strategy used free text and Medical Subject Headings (MeSH) terms including “neoplasms,” “cardiovascular disease,” “cardio-oncology,” and “aged,” as well as keyword combinations such “comorbidities in geriatric patients” and “old patient neoplasia”.

Titles and abstracts were reviewed independently by two researchers. Studies were excluded if they were clinical trial protocols, conference abstracts, non-peer-reviewed, articles, unavailable in full text, non-human studies, or written in languages unfamiliar to the authors. The inclusion criteria were studies on older adult patients (aged 65 years or above) of any gender, cancer type, or ethnicity. Eligible studies also needed to address one or more of the following: shared pathophysiological mechanisms of cancer and cardiovascular disease, decision-making in cancer care, cardiotoxicity, or shared decision-making processes in oncology. Data analysis was performed through data familiarization, and the findings are presented to address the main research question.

This review investigates the bidirectional relationship between cardiovascular health and oncology in older adults, specifically: (1) shared pathophysiological pathways; (2) the influence of baseline CV comorbidities on cancer trajectories and clinical decisions; (3) the cardiotoxic profiles and documented CV impacts of antineoplastic agents; and (4) prevention, monitoring and outcomes in the geriatric population.

This study employed a narrative synthesis approach rather than a systematic review; consequently, formal quality assessment and quantitative meta-analysis were not performed.

## 3. Results and Discussion

### 3.1. Epidemiology

The definition of an older person may vary across cultures, health systems, and social programs; however, the World Health Organization (WHO—www.who.int) and the United Nations (UN) define “older adults” as individuals aged 60 years and above. In 2020, the global population aged 60 and over exceeded the number of children under 5 years of age, and by 2050, it is estimated that people over 60 years will comprise approximately 22% of the world’s population [4].

Cardiovascular disease and cancer are the two leading causes of morbidity and mortality in older adults worldwide. With the global aging of the population, the number of individuals living with both conditions is rapidly increasing. The Satariano–Ragland score and Charlson Comorbidity Index are useful for evaluating the survival of older oncology patients and individualizing treatment protocols [5].

Approximately 60% of all new cancer diagnoses occur in individuals aged 65 years and older, and the majority of cancer-related deaths are also reported in this age group [6]. On 1 January 2025, according to Eurostat, people aged 65 and over made up 22% of the total European Union (EU) population, following an increase between 2015 and 2025. Likewise, cardiovascular disease remains the most common chronic condition in older adults, with nearly 70% of individuals aged 65 years and above affected by hypertension, coronary artery disease, or heart failure [7]. According to recent CDC data (August 2021–August 2023), 71.6% of U.S. adults aged 60 and older have hypertension. In the EU, prevalence is 63% for those aged 65–79 and 75% for those over 80. The prevalence of dyslipidemia among older adults in Europe is estimated at 76%. Nevertheless, the condition remains significantly underdiagnosed [8]. In 2024, approximately 23.7% of the global population aged 65–99 years was living with diabetes, with the highest prevalence in Middle East and North Africa [9]. In older cancer survivors, CVD is a major cause of non-cancer-related death. A large-scale retrospective study involving over 1.1 million older adult cancer patients found that cardiovascular mortality exceeded cancer mortality in certain long-term survivors, particularly those with early-stage tumors [10].

Cardiovascular comorbidities are particularly prevalent in certain cancer populations. Among geriatric breast cancer patients with hormone receptor-positive, HER2-negative metastatic disease, 61.7% had at least one cardiovascular comorbidity at the time of diagnosis, including hypertension, dyslipidemia, or diabetes [11]. In lung cancer patients aged over 70 years, more than 50% had pre-existing CVD, which significantly impacted their overall survival [12].

Another recent population-based study found that geriatric cancer patients are more likely to have cardiovascular comorbidities compared to older individuals without cancer, suggesting a higher vulnerability and complexity in this subgroup [13].

Epidemiological research among patients with neoplasia aged 60 and older revealed differences in treatment and outcomes in comparison to younger patients, with worse prognosis for those aged over 80 years [14].

A key challenge in understanding the impact of the interplay between CVD and cancer is that older patients with significant cardiovascular disease are often excluded from clinical cancer trials, resulting in a lack of evidence-based guidelines for this specific population.

### 3.2. Pathophysiological Mechanisms Shared by Cancer and Cardiovascular Disease

The intersection between cancer and CVD is not merely coincidental; multiple overlapping biological mechanisms link the two. In older patients, this interplay is further accentuated by aging-related processes, reduced physiological reserve, and cumulative lifetime exposures. This section discusses the principal pathways through which neoplastic disease—including its therapy—and CVD influence each other.

#### 3.2.1. Chronic Inflammation

As individuals age, a persistent, low-grade inflammatory state develops—sometimes referred to as “inflammaging” [15]. Pro-inflammatory markers such as IL-1, IL-6, IL-8, IL-13, C-reactive protein (CRP), tumor necrosis factor-α (TNF-α), interferon-α (IFNα), interferon-β (IFNβ), transforming growth factor-β (TGF-β), serum amyloid A, and CA125 are found at elevated levels in older patients [16,17,18]. IL-1β and IL-6 have been implicated in the progression of heart failure, arterial thrombosis and cancer metastasis, as elevated levels of these pro-inflammatory cytokines are associated with adverse cardiovascular outcomes and pro-tumorigenic signaling pathways [19]. Studies investigating clinical factors and molecular biomarkers in ovarian tumors have identified pathways involved in cell proliferation, inflammation, and tissue remodeling—mechanisms that are also implicated in cardiovascular disease pathophysiology [20].

S100A8/A9 (also known as calprotectin) is a calcium-binding heterodimer protein complex primarily produced by neutrophils and monocytes, acting as a crucial mediator in inflammation, infection, and tumor development. Extracellular S100A8/9 released by cancer cells promotes pro-inflammatory responses and immune suppression. While S100A8/A9 typically drives tumorigenesis, tumor aggressiveness, and metastasis—often contributing to immunotherapy resistance—it can also be tumor-suppressive in specific contexts, such as head and neck squamous cell carcinoma, depending on the mediated signaling pathways.

Furthermore, S100A8/9 can induce abnormal oxidative metabolism leading to the increased production of reactive oxygen species (ROS) [21,22]. It is also involved in several CVDs, such as atherosclerosis, where it increases endothelial permeability and triggers plaque instability and vascular smooth muscle cell proliferation. Additionally, it is implicated in aortic dissection, aortic aneurysm myocardial infarction, acute hypertension, heart failure, and myocarditis [23]. Given the dual roles of S100A9, its anti-inflammatory role beyond its pro-inflammatory effects necessitate defining a precise therapeutic window, during the acute inflammatory phase, to optimize S100A9 blockade as a safe immunomodulatory treatment strategy.

NLRP3 inflammasomes are strong inflammatory structures that can be activated by S100A8, S100A9, tobacco, cholesterol crystals, ATP, oxidized low-density lipoprotein, reactive oxygen species, potassium efflux, and lysosomal damage, as well as pathogen-associated molecular patterns, such as coxsackievirus B3 and human papillomavirus, covering a broad spectrum of triggers present in or provoking both CVD/heart failure and cancer. NLRP3, IL-1β, and IL-6 contribute to cancer progression through various pathways including the promotion of tumorigenesis, angiogenesis, immunosuppression, and metastasis, though NLRP3 can be tumor-suppressive depending on the cancer type (e.g., in colitis-associated colorectal cancer). The NLRP3 inflammasome and its product IL-1β are important mediators in the development, destabilization, and progression of atherosclerotic plaques; they increase the endothelial permeability and induce the proliferation, migration, and calcification of vascular smooth muscle cells [24,25].

#### 3.2.2. Microbial Dysbiosis

Inflammation serves as a defense mechanism against pathogens and contributes to tissue repairs; however, persistent inflammation in the absence of infection can be detrimental. Chronic inflammation in aging is influenced by chronic viral infections (e.g., CMV, HPV, and hepatitis), bacterial infections (e.g., periodontitis, *Helicobacter pylori*, *Schistosoma*), and alterations in gut microbiota and permeability, as well as metabolic disturbances such as obesity and hypernutrition (metabolic inflammation) [26,27,28]. This contributes to atherogenesis, endothelial dysfunction, and cancer promotion via cytokines, growth factors, and chemokines [29].

Gut microbial dysbiosis is increasingly recognized as a pivotal factor in the pathogenesis of cardiovascular diseases (CVDs), such as atherosclerosis, hypertension, and heart failure. The microbiota-dependent metabolism of dietary precursors generates key bioactive metabolites—including trimethylamine N-oxide (TMAO), phenylacetylglutamine (PAGln), bile acids (BAs), and short-chain fatty acids (SCFAs)—which serve as systemic signaling molecules. Clinical evidence suggests that circulating concentrations of these metabolites correlate with CVD risk and modulate essential homeostatic processes, including hemodynamic regulation, systemic inflammation, lipid metabolism, and vascular endothelial integrity [30]. The microbiota can promote or inhibit cancer through several key molecular pathways: (1) DNA damage: some bacteria produce toxins that induce direct host DNA damage (e.g., *Escherichia coli*), while others cause indirect damage by generating ROS (e.g., *Enterococcus faecalis*); (2) inflammatory signaling: pathogenic species like *Fusobacterium nucleatum* and enterotoxigenic *Bacteroides fragilis* (ETBF) trigger pro-inflammatory pathways (e.g., NF-κB, STAT3, and Wnt/β-catenin), creating an immunosuppressive tumor microenvironment (TME) that fosters malignant growth; and (3) metabolic reprogramming: microbial metabolites such SCFAs exert anti-tumor effects by inhibiting histone deacetylases (HDACs), which promotes apoptosis and maintains gut barrier integrity, while secondary bile acids can act as tumor promoters [31].

#### 3.2.3. Oxidative Stress

Excess ROS can damage DNA, lipids, and proteins, thereby promoting mutagenesis, endothelial injury, and cardiomyocyte death. Similar oxidative damage is also induced by antineoplastic agents, including anthracyclines, tyrosine kinase inhibitors, and antimetabolites [32,33,34]. Oxidative stress damages mitochondria, leading to cardiac hypertrophy and apoptosis, while in cancer, it contributes to maintaining pro-tumorigenic metabolic states [35]. Many chemotherapeutic agents induce mitochondrial dysfunction by damaging mitochondrial DNA or impairing electron transport chain activity, thus reducing ATP production and increasing ROS generation. Cardiomyocytes, which are highly dependent on mitochondrial energy, are particularly vulnerable to these effects. Aging and cumulative chronic genotoxic stress—from endogenous metabolism or various exposures to radiation, chemotherapy, or toxins—lead to DNA damage and telomere shortening. These processes are implicated in both increased cancer risk and cardiovascular aging. When DNA repair mechanisms fail, cells are programmed to activate apoptotic or senescence pathways to prevent the replication of a damaged genome [36].

Epigenetic changes—such as DNA methylation, histone changes, microRNA shifts—can lead to genomic instability, oncogene activation, cancer progression, metastasis, and drug resistance. To target these shifts, epigenetic therapy has been integrated into various cancer treatment regimens in order to prevent drug resistance and enhance the anticancer immune response [37]. Biological age assessed by DNA methylation patterns may correlate with cancer risk and could serve as an early diagnostic marker [38].

#### 3.2.4. Metabolic Dysregulation and Behavioral Factors

Obesity, hyperinsulinemia, dyslipidemia and metabolic syndrome are well-known risk factors for both cancer and CVD. Adipose tissue functions as a dynamic endocrine organ that secretes estrogen and key adipokines, such as adiponectin and leptin, which modulate cellular proliferation and elevate the risk of hormone-dependent malignancies. Beyond metabolic regulation, adipocytes and resident immune cells produce a suite of bioactive proteins and pro-inflammatory cytokines—including TNF-α, IL-6, and PAI-1—facilitating angiogenesis, atherosclerosis, and tumorigenesis. Thus, obesity is associated with chronic inflammation, leading to DNA damage and mutagenesis. Leptin dysregulation increases endothelial cell migration, VEGF, and pro-inflammatory cytokines. Furthermore, leptin causes endothelial dysfunction and the osteoblastic differentiation of vascular cells, promotes platelet aggregation, and triggers cholesterol accumulation in macrophages under hyperglycemic conditions, leading to CVD [39].

Several alterations, including endothelial damage, reduced nitric oxide bioavailability, and increased vascular stiffness commonly observed in aging, hypertension, and diabetes, promote atherosclerosis. These may also impair tumor perfusion control or microenvironmental regulation, contributing to cancer cell death. Platinum-based compounds and certain targeted therapies can exacerbate endothelial injury, while other treatments can cause vasospasm (e.g., 5-FU) or induce a prothrombotic state, worsening the overall prognosis [40,41,42]. Moreover, elevated D-dimer levels reflect the interplay between inflammation, endothelial injury, and hypercoagulability, processes central to both cancer progression and cardiovascular disease, and may serve as biomarkers of disease severity in complex systemic conditions [43].

A sedentary lifestyle, defined as any waking activity characterized by an energy expenditure less ≤ 1.5 metabolic equivalent tasks (METs) performed in a sitting or reclining position, is a significant, independent risk factor for both cancer and CVD in older adults. Prolonged sedentary behavior in older adults is linked to a 30% increase risk of fatal and non-fatal CVD due to endothelial dysfunction, dyslipidemia, hypertension and left ventricle dysfunction [44]. High sedentary time is associated with a 13% to 20% higher risk of cancer incidence, particularly colon, endometrial, ovarian, and prostate cancers. Inactivity causes hyperinsulinemia and alteration in the insulin-like growth factor (IGF) axis, which can stimulate tumoral cell proliferation, maintain low-grade chronic inflammation, alter sex hormone levels and increase adiposity [45]. As adults age, they often experience a natural decline in mobility, which, when combined with sedentary behaviors, accelerates sarcopenia (loss of muscle mass) and frailty. Older patients often have multiple comorbidities that reduce their mobility, forcing them to a sedentary lifestyle.

Smoking induces DNA damage that accumulates over a lifetime. As individuals age, their immune systems become less effective at eliminating cancer cells—a process further compromised by smoking. Additionally, smoking is a primary cause of CVD, as it promotes endothelial dysfunction, accelerates atherosclerosis, triggers hypercoagulability, and induces lipid abnormalities.

#### 3.2.5. Platelet Activation and Hemostatic Dysfunction

Cancer and CVD share a prothrombotic state. Platelets play a key role in inflammation, immune modulation, and tumor angiogenesis, while simultaneously contributing to thrombus formation and vascular occlusion. Indeed, cancer-associated thrombosis is the second most common cause of death in oncological patients. The interaction of platelet with circulating tumor cells is required for successful hematogenous metastasis. Platelets are highly versatile, capable of interacting with many types of cells within the vasculature; their capacity to survive and function within the tumor microenvironment make them a crucial mediator of tumor progression and metastasis [46].

Beyond thrombosis, platelets have multiple functions, playing important roles in atherosclerosis, cardiac fibrosis, airway hyperresponsiveness, airway wall remodeling, intestinal colitis, and tumor metastasis. In atherosclerosis, vascular endothelial dysfunction triggers the rapid adhesion of activated platelets, a pivotal event that initiates and propagates vascular inflammation. This contributes to angiogenesis, the secretion of pro-inflammatory chemokines and cytokines, and the alteration of the vascular microenvironment, promoting further plaque formation [47,48].

Some of the most common pathophysiological mechanisms of CVD and cancer are summarized in Table 1.

### 3.3. Clinical Implications

Older patients with concomitant cardiovascular disease (CVD) and cancer, including cancer survivors, experience significantly worse clinical outcomes than those affected by only one of these conditions. These comorbidities substantially influence survival, treatment decisions, quality of life, and the risk of adverse events. Aging, obesity, smoking, diabetes mellitus, and arterial hypertension are common risk factors that can independently and synergistically worsen the prognosis of these patients. Therefore, cancer survivors with pre-existing CVD have poorer outcomes with five-year survival rates of approximately 75% compared with 87% in those without CVD. In addition, the potential cardiotoxicity of oncologic therapies must be carefully considered when managing these patients. While advanced age is a risk factor for malignancy, cancer and oncological treatments accelerate cellular senescence [50].

Several studies and meta-analyses have demonstrated that patients with specific cardiovascular diseases are at an increased risk of developing cancer [51,52,53]. There is a bidirectional relationship between cancer and atrial fibrillation (AF), one of the most frequent arrhythmias in adult patients. Both conditions share common risks factors such as aging, hypertension, obesity, diabetes, and smoking. Oncology patients have a higher risk of AF due to the direct cardiotoxic effects of the cancer therapies. Inflammation may promote both structural and atrial electrical remodeling, contributing to the development of AF [54]. In addition, the hypercoagulable state associated with cancer may trigger atrial fibrillation through pulmonary microembolism and subsequent atrial strain [55,56]. The management of AF in oncology patients is challenging due to the high risk of both thromboembolism and bleeding, as well as drug interactions between anticoagulants and cancer therapies.

Radiotherapy, particularly chest irradiation used in the treatment of Hodgkin lymphoma, non-Hodgkin lymphoma, and breast and lung cancers, causes damage to vascular walls, induces inflammation and fibrosis, and accelerates the development of atherosclerosis [57,58]. Related cardiovascular toxicity usually develops 5–10 years after the initial treatment and may result in coronary artery disease, heart failure, or peripheral artery disease, with an incidence up to sixfold higher than in the general population. This risk depends on the radiation dose delivered to the cardiovascular tissues, the concomitant use of other oncological therapies, and the presence of pre-existing cardiovascular comorbidities [59,60]. In addition, several chemotherapeutic agents, including anthracyclines and cyclophosphamide, disrupt intracellular calcium homeostasis, leading to calcium overload, impaired myocardial contractility, increased arrhythmogenic potential and cardiomyocyte apoptosis [61,62].

### 3.4. Mortality and Competing Risks

In long-term cancer survivors, cardiovascular mortality may surpass cancer-related mortality over time as a consequence of treatment toxicity, age-related frailty, and shared risk factors. This shift in the primary cause of death underscores the necessity of multidisciplinary management and has driven the emergence of cardio-oncology as a specialized field. Multiple studies across different cancer types and populations have consistently reached similar conclusions. For instance, a mixed-race population-based study of patients with esophageal cancer showed that, five years after diagnosis, the majority of deaths were attributable to cancer causes, but over 10 years, 59% of patients died from noncancer causes—particularly cardiovascular (43%) and respiratory diseases [63]. Similarly, among breast cancer patients treated with chemotherapy, those over 76 years of age experienced significantly more cardiovascular events compared to those ages 66–75, highlighting age-related vulnerability to treatment-induced cardiac complications [64]. A large population-based study of older bladder cancer patients demonstrated that cardiovascular disease was the leading cause of mortality (70%) 10–15 years after diagnosis, especially among patients with pre-existing cardiovascular disease [65].

Mortality rates may vary across different regions. A population-based study of CVD mortality risk in U.S. patients with cancer showed that the highest rates of CVD-related deaths occurred in those with bladder, larynx, prostate, uterine, colorectal, and breast cancer. Conversely, patients with lung, brain, liver, gallbladder, stomach, pancreas, esophageal, and ovarian cancer, as well as multiple myeloma, were least likely to die from CVD. Certain malignancies—such as soft tissue, nasopharyngeal, anal, oropharyngeal, colorectal, kidney and cervical cancers, as well as non-Hodgkin’s lymphoma—appear to have higher CVD-related mortality, likely due to their comparatively higher long-term survival rates. Compared with the general population, cancer patients diagnosed after the age of 60 demonstrated a decreasing relative risk of CVD death with increasing age, which may reflect the higher baseline incidence of CVD in the general older population [10].

In patients with metastatic cancer, mortality due to cancer is substantially higher, particularly among patients aged over 60. Among non-cancer deaths in this population, CVD was the most common cause. Patients with metastatic cancer have a tenfold higher risk of dying from heart or cerebrovascular disease within the first year after diagnosis compared with the general population. This elevated risk may reflect the increased cardiotoxic potential of complex oncological treatments, especially in individuals with pre-existing cardiovascular disease [66].

### 3.5. Morbidity, Complications, and Quality of Life

Cardiovascular comorbidities increase the likelihood of treatment complications. For example, older patients with pre-existing heart failure are often ineligible for standard chemotherapy (e.g., anthracyclines), which can reduce treatment efficacy and worsen oncological outcomes [67]. Drugs such as anthracyclines, human epidermal receptor 2 (HER2)-targeted therapies, vascular endothelial growth factor (VEGF) inhibitors, proteasome inhibitors (PI), immune checkpoint inhibitors (ICI), tumor-infiltrating lymphocyte (TIL) therapies, and chimeric antigen receptor T cell (CAR-T) therapies require rigorous cardiac monitoring. Because these are among the most cardiotoxic oncological agents, treatment must be paused or discontinued if cardiac damage occurs. The higher survival rates in cancer patients, due to the development of new drugs over time, have been accompanied by some cardiovascular complications, as summarized in Table 2 [68].

A baseline cardiovascular assessment should be performed before initiating oncological treatment. Cardiovascular risk scores combined with the patient’s medical history, lifestyle factors, and clinical examination can help detect previously undiagnosed conditions such as heart failure, valvular disease, pericardial disease, arrhythmias, or venous thromboembolism. For patients with pre-existing CVD, the cardiovascular evaluation may be completed with stress echocardiography, nuclear perfusion imaging, coronary computed tomography angiography, or cardiac magnetic resonance. Standard baseline cardiovascular assessment should include a 12-lead ECG, transthoracic echocardiography, and cardiac serum biomarkers such as natriuretic peptide (BNP, NT-proBNP), and cardiac troponin I or T [68,69].

The coexistence of CVD and cancer negatively affects physical function, increases fatigue and reduces mobility and independence, ultimately worsening quality of life. Coexisting conditions and concomitant medication may interact adversely with cancer medication. Many older cancer patients also experience functional decline with impaired hearing, vision, balance, strength, or memory, which complicates self-care. Some patients live alone without adequate social or financial support, and are prone to depression, anxiety and fear of being a burden to their families. Previously, palliation was often the only option for frail, older adults with multiple comorbidities. Today, comprehensive care is increasingly applied, involving multidisciplinary teams that take care of the physical, emotional, and social needs of geriatric oncological patients, often prioritizing quality of life over the mere extension of survival [70,71,72].

### 3.6. Impact on Oncologic Treatment Decisions

When deciding to treat an older oncologic patient, several factors must be considered, including comorbidities, frailty and cognitive ability. These are usually assessed using tools such as the Mini-Mental State Examination (MMSE) score and Montreal Cognitive Assessment (MoCA), as well as medical interactions among multiple concomitant medications. Each of these factors is associated with poorer clinical outcomes, increased treatment-related toxicity, reduced survival, and increased rates of hospital readmission [73].

Cardiovascular status plays a critical role in determining the type and intensity of cancer treatment. In older patients with cardiovascular comorbidities, oncologists may opt for less aggressive therapies or reduced doses to minimize the risk of cardiac complications. Furthermore, drug–drug interactions and polypharmacy in older patients complicate treatment planning, requiring close collaboration between oncologists, cardiologists and geriatricians [74].

To evaluate a patient’s frailty and functional status, it is important to assess their physical condition and the presence of geriatric syndromes in order to determine treatment tolerance and prognosis and to maintain an adequate quality of life. Geriatric syndromes include conditions such as falls, neurocognitive disorders, delirium, urinary incontinence, difficulty with daily activities, chronic pain, dizziness or syncope, pressure ulcers, malnutrition, and sleep disorders [75].

A Comprehensive Geriatric Assessment (CGA) is essential for evaluating frailty, cognitive function, polypharmacy, and nutritional, psychological, social, and environmental status, as well as functional independence. This assessment helps predict treatment tolerance and enables risk stratification, thereby preventing under- or overtreatment [76]. Identifying high-risk patients before treatment initiation allows for early intervention and dose adjustment to prevent complications. Ultimately, age and comorbidities significantly influence tumor board decisions and are frequently associated with lower adherence to standard ESMO guideline recommendations [77].

### 3.7. Prevention, Monitoring, and Rehabilitation

The adaptation of cancer treatment should be performed by the oncological team. In patients at high cardiovascular risk, less cardiotoxic agents may be preferred, such as epirubicin instead of doxorubicin or liposomal anthracyclines [25,78,79,80]. Several cardioprotective strategies have emerged to mitigate cardiac risk, including the use of dexrazoxane, dose reductions, continuous infusion, and the careful monitoring of left ventricular function [81]. Angiotensin-converting enzyme (ACE) inhibitors, angiotensin receptor blockers (ARBs) and beta blockers are used to manage and potentially prevent cardiac dysfunction, particularly in high-risk patients [82,83,84]. Statins may also offer protective effects against cardiotoxicity due to their anti-inflammatory and antioxidative properties [85,86]. Furthermore, recent studies on sodium–glucose cotransporter 2 (SGLT2) inhibitors have revealed promising data regarding their potential role in preventing cardiovascular toxicity. Treatment plans should be individualized, balancing cancer prognosis with cardiovascular safety and life expectancy.

Preventive strategies include strict control of cardiovascular risk factors through smoking cessation, dietary modifications, weight reduction, and physical activity tailored to the patient’s condition. The regular monitoring of cardiac function is recommended during and after cancer therapy, especially for high-risk patients, as indicated by the cardio-oncology guidelines.

Cardiac rehabilitation and specialized cardio-oncology programs have shown promising results in improving patient outcomes. Patients who complete structured rehabilitation programs often experience better survival rates, improved physical fitness and an enhanced quality of life. For frail older patients or those living in remote areas, telemedicine and home-based rehabilitation may serve as viable alternatives to in-person care.

## 4. Conclusions

The coexistence of CVD and cancer in older patients represents a significant clinical challenge, supported by epidemiological data showing a high prevalence of both conditions and frequent co-occurrence in this population. This association is driven by overlapping risk factors, shared pathophysiological pathways, and increased treatment complexity. Aging contributes to the vulnerability of both systems, while cancer therapies may further amplify cardiovascular risk through mechanisms such as oxidative stress, cellular senescence, and inflammation, as discussed in this review.

Clinical evidence indicates that cardiovascular comorbidities are associated with increased mortality, reduced treatment tolerance, and a higher incidence of therapy-related complications in cancer patients. Additionally, several oncologic therapies have been shown to induce or exacerbate cardiovascular dysfunction, further complicating management in older individuals. In this regard, clinicians must recognize the dual burden of cancer and CVD when treating older patients, carefully balancing oncological efficacy with cardiovascular safety while respecting patients’ preferences.

Early risk assessment, interdisciplinary collaboration, and individualized treatment strategies, including cardioprotective interventions, are essential to improve outcomes and preserve quality of life in this complex population. Given the high incidence of cardiovascular events observed in cancer survivors, particularly in long-term follow-up, systematic monitoring and the early identification of high-risk patients are necessary components of clinical care. Further research is needed to provide robust, evidence-based recommendations tailored to older patients, who remain underrepresented in clinical trials.

## Figures and Tables

**Table 1:** Shared pathophysiological mechanisms of cardiovascular disease and cancer.

Mechanism	Molecular Drivers	Role in CVD	Role in Cancer
Chronic Inflammation	S100A8/A9, NLRP3, IL-1β, IL6, TNF α	Triggers endothelial dysfunction, foam cell formation, and plaque instability. Persistent inflammation leads to myocardial remodeling and heart failure [24].	Creates an immunosuppressive microenvironment that helps tumors evade detection, promotes angiogenesis and metastasis [22,23].
Microbial Dysbiosis	TMAO, SCFAs	Gut imbalance produces metabolites like TMAO that directly promote arterial plaques and worsen cardiac fibrosis [29,30].	Pathogenic bacteria can activate several pathways, promoting tumor growth, especially in the colon [31].
Oxidative Stress	ROS	Excess reactive oxygen species (ROS) damage mitochondrial DNA in cardiomyocytes, causing cell death (apoptosis) and worsening HF [32,33,34,35].	Causes DNA mutations. ROS activate pathways that drive tumor proliferation and chemoresistance [36,37,38].
Metabolic Dysregulation	Adipokines, insulin, insulin-like growth factor	Disrupted fatty acid metabolism leads to elevated lipids (atherosclerosis) and insufficient energy (ATP) for heart function [39].	Cancer cells undergo metabolic reprogramming to fuel rapid biomass synthesis and proliferation.
Platelet activation and hemostatic dysfunction	ADP, TXA2, IL-1β, growth factors, angiogenetic factors	Vascular endothelial dysfunction triggers rapid adhesion of activated platelets, promoting atherosclerosis [47,48].	Platelets play a key role in inflammation, immune modulation, and tumor angiogenesis [46].
Neuro-hormonal Activation	RAAS (Angiotensin II), Adrenergic (SNS)	Chronic activation of the RAAS system leads to hypertension, cardiac remodeling, and fibrosis.	Adrenergic and RAAS signaling can promote tumor angiogenesis, tissue invasion, metastasis and resistance to apoptosis [49].

**Table 2:** Cardiovascular complications during cancer therapy.

	Cardiomyopathy Heart Failure	Myocardial Ischemia	Myocarditis	Hypertension	Arrhythmias	QTc Prolongation	Pulmonary Hypertension	Venous Thromboembolism	Peripheral Arterial Toxicity	Pericardial Effusion	Pleural Effusion
Anthracycline	X (dilatative)										
HER2-targeted therapies	X										
Fluoropyrimidines	X Takotsubo	X	X	X	X				X		
VEGFi	X	X		X	X	X		X	X		
Multitargeted kinase inhibitors targeting BCR-ABL	X			X	X		X		X	X	X
Bruton tyrosine kinase	X			X	X (AF)						
Alkylating agents	X (HF)				X (AF)						
Proteasome inhibitors	X (HF)			X	X (AF)		X	X			
Monoclonal antibodies	X (HF)			X	X (AF)						
Rapidly accelerated fibrosarcoma (RAF) inhibitors	X (HF)			X	X	X		X			
Mitogen-activated extracellular signal-regulated kinase (MEK) inhibitors	X (HF)			X							
Immune checkpoint inhibitors	X	X	X		X (AVB)		X		X (ischemic stroke)	X	
Androgen deprivation therapy	X	X		X							
Aromatase inhibitors	X	X		X							
Cyclin-dependent kinase 4/6 inhibitors						X					
Anaplastic lymphoma kinase (ALK) inhibitor				X	X (SVE)	X					
Epidermal growth factor receptor (EGFR)inhibitor	X				X (AF)	X		X			
Chimeric antigen receptor T cell (CAR-T cell)	X (HF, Takotsubo)				X					X	
Radiotherapy	X	X	X						X	X	
Platinum-containing chemotherapy		X						X	X		

AF—atrial fibrillation, AVB—atrio-ventricular block, HF—heart failure, SV—supraventricular extrasystoles, X—marks the existence of that pathology.

## Data Availability

All data is contained within the article.

## Abbreviations

The following abbreviations are used in this manuscript:

ACE	angiotensin-converting enzyme inhibitors
AF	atrial fibrillation
ARB	angiotensin receptor blockers
AVB	atrio-ventricular block
BA	bile acids
CAR-T	therapies and chimeric antigen receptor T cell therapies
CMV	cytomegalovirus
CRP	C-reactive protein
CVD	cardiovascular disease
HDAC	histone deacetylases
HER	human epidermal receptor
HF	heart failure
HPV	human papillomavirus
ICI	immune checkpoint inhibitors
PAGl	phenylacetylglutamine
PI	proteasome inhibitors
ROS	reactive oxygen species
TGF	transforming growth factor
TMAO	trimethylamine N-oxide
TME	tumor microenvironment
TNF	tumor necrosis factor
TIL	tumor-infiltrating lymphocytes
RAAS	renin angiotensin aldosterone system
SCFA	short-chain fatty acids
UN	United Nations
VEGF	vascular endothelial growth factor
WHO	World Health Organization
